# The global dynamics of RNA stability orchestrates responses to cellular activation

**DOI:** 10.1186/1741-7007-8-95

**Published:** 2010-07-08

**Authors:** Jack D Keene

**Affiliations:** 1 Department of Molecular Genetics and Microbiology, Duke University Medical Center, Durham, NC 27710, USA

## Abstract

Transcriptomics is used to quantify changes in accumulated levels of mRNAs following cellular activation. These changes arise from the opposing fluxes of transcription and mRNA decay, both of which affect the functional dynamics of global gene expression. A study published recently in *BMC Genomics *focuses on the contribution made by mRNA stability in shaping the kinetics of gene responses in mammalian cells.

See research article http://www.biomedcentral.com/1471-2164/11/259

## Commentary

**Figure 1:**
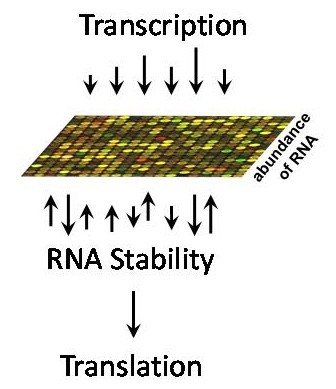
**The abundance of each mRNA detected in a microarray assay is determined by its rates of transcription and decay**. Transcriptional and post-transcriptional events operate in tandem to dictate the level of each mRNA at a given time point, and the kinetics of gene expression under different conditions. Several studies and review articles, some of which are cited in this article, have demonstrated that mRNAs encoding functionally related proteins can change stability in concert with one another following cellular activation.

A commonly overlooked fact regarding microarray-derived as well as high-throughput sequencing data is that the accumulated (so called steady-state) levels of mRNAs depend on rates of RNA transcription and degradation at the time of RNA extraction (Figure 1). While this fact has become increasingly evident among researchers who are interested in the underlying mechanisms of gene expression, those focused on transcription *per se *often interpret their data while assuming that changes in mRNA levels are due to the activation or repression of transcription alone.

It is therefore useful to have this assumption further challenged by Agami and co-workers (Elkon *et al*.) [1], who examined on a global scale the role of mRNA stability in shaping the kinetics of gene responses. RNA decay is now a rapidly growing field, but only recently have investigators begun to consider that global changes in mRNA stability can indicate systematic coordination across functionally related transcripts as governed by *trans*-acting factors [2]. Indeed, several studies over the past 10 years have found that specific groups of transcripts can coordinately change their stability in response to cellular signals - and that this is a property of most organisms. This tenet of the posttranscriptional RNA operon/regulon model of coordinated gene expression is best illustrated in studies of the immune system of vertebrates and in model organisms such as yeast and trypanosomes (reviewed in [3-7]).

## Global kinetics of mRNA transcription and stability

Elkon *et al. *[1] examined published gene-expression profiles that had been generated by stimulation of T cells with interleukin 2 (IL-2), and evaluated these data in relation to other published data comprising a global atlas of mRNA stability [8]. The mRNAs induced in response to IL2 grouped as three clusters, the early, intermediate and late-responding gene groups, and there was a striking correlation between the response time post IL2 stimulation and the intrinsic stability of the mRNA. The authors went on to evaluate nine more time-course gene expression datasets against the mRNA stability data and thus demonstrated that this correlation held generally for a variety of mammalian cell responses to different physiological stimuli. These and other studies of orchestrated kinetics of functionally related genes during an inflammatory response to cytokine treatment led to similar interpretations [3]. The rapidly responsive (early) populations contained mRNAs that were less stable and the late responding populations contained RNAs that were more stable. This finding fits the predictions of a previously published kinetic model [6] and is consistent with a long-held supposition that genes encoding mRNAs with short half-lives are best suited to respond quickly to transcriptional activation or suppression [2]. Thus, the intrinsic stability of the mRNA would be expected to affect the rate of induction as well as the suppression of mRNA levels because transcripts with short half-lives can respond more rapidly than stable transcripts to increases or decreases in transcription rates.

However, Elkon *et al. *[1] found that this relationship broke down for those genes that were being rapidly suppressed as in at least half of the datasets examined there was no correlation between the rapidity of downregulation and the inherent instability of the decreasing mRNAs. In many cases, there was a rapid downregulation of otherwise stable mRNAs, leading the authors to logically propose that *trans*-acting RNA-binding proteins and/or specific microRNAs were probably activated during suppression to help destabilize these otherwise stable subsets of mRNAs.

Observations that similarly support a coordinated regulation of mRNA stability were obtained in recent studies by Shalem *et al*. using *Saccharomyces cerevisiae *[9]. In this work, quantification of changes in the abundance of mRNAs during two conditions - induced oxidative stress and induced DNA damage - showed very different kinetic patterns that were analyzed for RNA synthesis and decay. Treatment with hydrogen peroxide resulted in a rapid but transient response as exemplified by the rise and fall of mRNAs encoding proteasomal components. On the other hand, treatment with the DNA mutagen methyl methanesulfonate resulted in an increasing accumulation of proteasomal mRNAs over the same time period, representative of a more enduring global response. When Shalem *et al*. [9] inhibited transcription in order to examine the contribution of RNA synthesis versus decay to the kinetics of the induced and suppressed genes on a global level, they concluded that during the transient response to oxidative stress, induced mRNAs became, on average, more destabilized while suppressed transcripts tended to become more stable. Thus, evidence of counteraction between synthesis and decay of functionally related mRNA subpopulations was proposed to facilitate these rapid responses. On the other hand, after DNA damage, there was a trend for the mRNAs that increased in abundance to become more stable, while repressed mRNAs tended to become less stable. In sum, these findings indicate a condition-specific regulation of mRNA stability and suggest that fast relaxation responses, such as those occurring after oxidative stress, may be accelerated by destabilization of induced gene transcripts and stabilization of the repressed mRNAs. In aggregate, data from this and other studies described here suggest that functionally related subsets of mRNAs can be modulated by mRNA turnover in a condition-specific manner and that these putative post-transcriptional RNA operons/regulons are probably coordinated by different combinations of *trans*-acting RNA-binding proteins and noncoding RNAs [2,3,5-7].

## Limitations and possible solutions

Agami and co-workers [1] concede that models and interpretations of global mRNA decay data can be easily oversimplified, and so new approaches will be needed to fully understand the dynamic properties of RNA regulation. Thus, one has to begin with simple assumptions and progressively refine the tools over time to address the complexity of this problem. Indeed, many issues surrounding experimental design and interpretation of mRNA stability data are not settled. The main one is the means by which transcription is experimentally inhibited, so that the data truly represent the balance between transcription and RNA stability without interference from chemical side-effects of the procedures. Conditional genetic switches are not available in mammalian cells for global analysis of mRNA decay, but temperature-sensitive mutants of RNA polymerase can be used with *Saccharomyces cerevisiae*, so that other cellular processes are minimally disturbed [6,9].

An alternative method of distinguishing the contributions of transcription and RNA stability to mRNA abundance in mammalian cells is nuclear run-on followed by microarray analysis or other high-throughput RNA quantification tools [2,10]. When applied to T-cell activation by Cheadle *et al*. [10] this approach revealed that at least 50% of the changes in accumulated mRNA levels detected by microarray were due to changes in RNA stability [10], and many workers believe that this is an underestimate. More recent use of thiolated nucleosides incorporated into nascent transcripts followed by capture of biotinylated transcripts has been used to quantify the transcriptional contribution to mRNA levels in response to cell activation before RNA processing can take place. Those changes in mRNA levels due to transcription can then be compared with RNA stability measurements of the population to evaluate the relative contributions of each process to the accumulated quantity of mRNA [8]. Interestingly, in the case of trypanosomes, one can avoid some of these technical concerns altogether while examining posttranscriptional regulation directly because transcription is not taking place during differentiation. Under these circumstances, co-regulated changes in mRNA levels that depend upon RNA stability alone can be quantified by microarray analysis. Several recent studies have demonstrated these coordinated changes of functionally related subsets of mRNAs during developmental stages of trypanosomes, consistent with the posttranscriptional operon/regulon model [5,11]. Given the large number of identified RNA-binding proteins in the trypanosome genome, ribonucleoprotein immunoprecipitation followed by microarray (RIP-chip) and related methods should be able to identify the combinations of factors responsible for coordinating trypanosome posttranscriptional RNA regulon decay dynamics. Indeed, determining the combinatorial interactions of RNA-binding proteins and noncoding RNAs that coordinate the dynamic changes of mRNA subpopulations following cellular activation will provide insights into mechanisms of coordinated global gene expression [2,4,7].

As shown in these studies, efforts to understand the underlying principles of mRNA stability are essential because the balance between transcription and decay influences most, if not all, responses of cells to endogenous and exogenous signals. However, the ability to correlate changes in RNA stability with changes in protein output is tenuous given the poor correlation between the transcriptomic profiles and proteomic profiles. Moreover, given that each mRNA has multiple copies and that some copies probably reside in different states of localization, stability and translation, one cannot yet infer how quantification of any one state reflects on the other states. Thus, mRNA decay curves represent an average of the stability changes of all copies of each mRNA regardless of their individual states. It is logical to expect that the regulatory factors associated with each individual mRNA copy dictate in aggregate and coordinate these observed patterns of mRNA stability. It will be important to identify these combinatorially associated factors during cell-activation events and to quantify the dynamic interactions among them that govern cell growth and development.
